# Anterior attentional system efficacy in Parkinson’s disease: a cross-sectional study in Poland

**DOI:** 10.3389/fnhum.2025.1695299

**Published:** 2025-11-13

**Authors:** Paulina Beata Golińska-Drobienko, Artur Sawicki, Łucja Bieleninik, Michał Schinwelski, Mariola Bidzan

**Affiliations:** 1 Faculty of Social Science, Institute of Psychology, University of Gdańsk, Gdańsk, Poland; 2 Neurocentrum-Miwomed, Gdańsk, Poland

**Keywords:** Parkinson’s disease, executive functions, attention, reaction times, cognitive impairment

## Abstract

**Introduction:**

Cognitive impairment is a prevalent nonmotor symptom of Parkinson’s disease (PD), significantly affecting patients’ quality of life. Considering the gap in understanding the relationship between cognitive impairments in Parkinson’s disease and executive function, this study aimed to investigate the association between three cognitive statutes: normal cognition condition (PD-NCC), mild cognitive impairment (PD-MCI), and mild dementia (PDD), and the performance of the Anterior Attentional System in individuals with Parkinson’s disease.

**Methods:**

This cross-sectional study included 96 participants with Parkinson’s disease (45 with PD-NCC, 39 with PD-MCI, and 12 with PDD) and 46 participants from control group, recruited between 2020 and 2023. MDS-UPDRS was used during the neurological examination. To assess cognitive status, we used: Mini-Mental State Examination, Californian Verbal Learning Test, Digit Span Test, Rey Complex Figure, and Trail Making Test (A and B form). The ROtman Baycrest Battery was employed to evaluate the Anterior Attentional System.

**Results:**

Participants with PDD exhibited significant impairments in the Anterior Attentional System. Energization impairment was observed in PD-MCI and PDD, though mildly. Monitoring and task-setting processes were notably impaired only in individuals with PDD, while these functions remained intact in those with PD-NCC and PD-MCI.

**Conclusion:**

The Anterior Attentional System is well-preserved in PD-NCC and PD-MCI however notably disturbed in PDD.

## 1 Introduction

By 2040, with the prevalence of Parkinson’s disease is projected to double, increasing from 6.9 to 14.2 million individuals (Dorsey and Bloem, 2018). One of the profound features of nonmotor symptoms in Parkinson’s disease is cognitive impairment (Aarsland et al., 2003; Bosboom et al., 2004; Litvan et al., 2012; Baiano et al., 2020). The identified mechanism of the disease—dopaminergic depletion—may contribute to reduced efficiency in information exchange between striatal and frontal areas (Cools et al., 2002; Leh et al., 2007; Bonelli and Cummings, 2008; Xu et al., 2016). Frontostriatal circuits, which are neural pathways connecting the frontal areas to the basal ganglia, play a critical role in mediating cognitive, motor, and behavioral processes (Zgaljardic et al., 2003; Sawamoto et al., 2008; Meier et al., 2013). This may account for the frequent observation of attentional and executive functions impairments in individuals with Parkinson’s disease, even from the very early stage of the disease, and executive problems are recognized as the most prevalent cognitive difficulty in Parkinson’s (Koerts et al., 2012; Dirnberger and Jahanshahi, 2013).

Furthermore, it has also been noticed that there are similarities between attentional and executive functions regarding their brain localization and process definitions (Fernandez-Duque and Posner, 2001). One of the main approaches is represented in the model described by Stuss et al. (1995, 2005) called the Anterior Attentional System (Stuss and Alexander, 2007). This system consists of three processes: energization, monitoring, and task-setting, each independently involving different parts of the frontal lobes (Stuss et al., 1995, 2005; Stuss and Alexander, 2007). However, the theoretical approach to organizing attentional processes, as represented by Stuss and colleagues, despite strong experimental evidence supporting prior authors’ assumptions, has not gained as much popularity as, for example, Posner’s theory. To our knowledge, the attentional processes, as tested by the Stuss methodology, have been studied in somatic diseases, such as end stage renal disease rather than in other neurological diseases (Harciarek et al., 2016).

Energization refers to the ability to maintain activation, which is defined as the optimal level of arousal over time. Previous studies have demonstrated that deficits in energization are observed in individuals with selective damages in medial frontal areas. Monitoring, also referred to as quality supervision, is the process responsible for evaluating the accuracy of responses. When performing tasks requiring monitoring, the right dorsolateral part of the frontal lobe is activated. Task-setting is involves establishing a criterion, following which a response might be given. This process is linked to the activation of the left dorsolateral part of the frontal lobe (Stuss et al., 2005). The ROtman–Baycrest Battery to Investigate Attention (ROBBIA) has been proposed as a reliable and valid tool for assessing these three components: energization, monitoring, and task-setting. The ROBBIA is based on reaction time measurement, and each process (energization, monitoring, and task-setting) is analyzed according to a specified formula regardless of Parkinsonian slowness (Stuss et al., 2005; Stuss and Alexander, 2007).

Summarizing, the Anterior Attentional System is one of several possible methodological approaches to organizing attentional processes and measuring them, as there are still many unknowns about attention. Considering previous scientific reports that have concentrated on attentional and executive processes in Parkinson’s disease, it has remained unclear so far how the three processes - energization, monitoring, and task-setting - proceed in individuals with Parkinson’s disease. However, performance in Anterior Attentional processes may differ due to heterogeneity of Parkinson’s disease.

To the best of our knowledge, no prior research has been conducted on Anterior Attentional System efficiency in individuals with Parkinson’s disease following ROBBIA methodology. However, it is essential to acknowledge that the performance of the Anterior Attentional System in individuals with Parkinson’s disease may vary depending on the severity and progression of the disease. The disease stage can also be defined differently due to the complexity of disease symptoms. Thus, the study aimed to assess the functioning of the Anterior Attentional System in individuals with Parkinson’s disease at different stages of disease progression. For this research, the disease progression was classified based on cognitive functioning – normal cognitive status (PD-NCC), mild cognitive impairment (PD-MCI), and mild dementia (PDD). We have decided to use the above disease progression definition because analyzing many highly correlated variables, including motor dysfunctions, might make this work hard to follow and out of scope. The following research questions have been formulated: (1) Are there differences in energization between PD-NCC, PD-MCI, PDD, and control group? (2) Are there differences in monitoring between PD-NCC, PD-MCI, PDD, and control group? (3) Are there differences in task-setting between PD-NCC, PD-MCI, PDD, and control groups?

## 2 Materials and methods

### 2.1 Study design

The project was designed as a cross-sectional study and received approval from the Ethics Committee of the University of Gdańsk, Poland (approval no. 45/2020). Participation in the study was voluntary and uncompensated. All participant provided informed consent after receiving detailed information about the research process, including the study’s methodology, coding and storing data, and the possibility of resigning at any time. Recruitment took place at medical facilities in Gdańsk and was carried out by neurologists specializing in Parkinson’s disease diagnosis and treatment. Upon providing written consent, participants received instructions regarding the two study stages: neurological examination (step I) and neuropsychological assessment (step II). Participants from the control group were recruited through public announcements. Research and recruitment procedures were feasibility-tested in advance (Golińska et al., 2021).

#### 2.1.1 Eligibility criteria

**a) The clinical group**

The clinical group consisted of individuals with idiopathic Parkinson’s disease. The inclusion criteria included (1) a diagnosis of idiopathic Parkinson’s disease confirmed by a neurologist based on the current International Classification of the Diseases 10th Revision (ICD-10 code: G20) (World Health Organization, 1993) (2) right-handedness; (3) being a native speaker in polish. The exclusion criteria were as follows: (1) advanced vision and hearing problems (preventing a standardized neuropsychological diagnosis); (2) major depressive episode and history of mental illness (including schizophrenia and bipolar disease; however individuals with mild depressive disorders were not excluded from the study); (3) addiction to alcohol or other psychoactive substances; (4) the history of ischemic or hemorrhagic stroke; (5) deep brain stimulation treatment; (6) other neurological diseases such as epilepsy. No restriction on age, gender, or education was applied. Participants were classified by two neurological experts into one of the three groups: (1) participants in normal cognitive condition (PD-NCC), (2) participants with mild cognitive impairment (PD-MCI), and (3) participants with mild dementia (PDD). The division was made in accordance with the criteria for assessing the cognitive functioning of individuals with Parkinson’s disease proposed by the Movement Disorders Society (Litvan et al., 2012).

**b) The control group (CG)**

Recruitment was conducted using the snowball sampling method. Participants in the clinical and control groups were matched based on education, gender, and age, with outliers excluded to ensure comparability. The inclusion criteria were as follows: (1) being native in the Polish language; (2) right-handedness. The exclusion criteria included: (1) a history of neurological diseases; (2) advanced vision and hearing problems; (3) diagnosed mental illness (however, participants who had been treated for depression in the past or were currently suffering from mild depressive disorders were not excluded from the study, the prevalence of depressive disorders is also high in the clinical group); (4) addiction to alcohol and other psychoactive substances; (5) a Mini-Mental State Examination (ref) score below 27 points.

### 2.2 Outcomes and measurement tools

**a) Step I - Neurological examination**

Neurologists conducted the neurological examination using the standard MDS-Unified Parkinson’s Disease Rating Scale (MDS-UPDRS) scale (scale I, II, III, IV) (Goetz et al., 2007). Furthermore, the structured medical interview was implemented to collect data about comorbidities and current Parkinson’s disease treatment, including the Levodopa Equivalent Daily Dose index (LEDD) (Julien et al., 2021).

**b) Neuropsychological assessment**

Neuropsychological assessment was divided into two steps: (1) standard neuropsychological assessment and (2) experimental anterior-attentional processes measurement.

b1. Standard neuropsychological assessment

Sociodemographic information was collected at the outset of the survey. Then, the tests were presented to each participant in the same order: Mini-Mental State Examination (MMSE) (Folstein et al., 1983), Californian Verbal Learning Test (CVLT) (Baños and Martin, 2002), Digit Span from Wechsler Intelligence Test WAIS-R (Brzeziński et al., 2004), Verbal Fluency Test; Trail Making Test from Halstead-Reitan Battery (TMT A and B) (Bowie and Harvey, 2006), Rey Osterrieth Complex Figure Test (ROCF) (Shin et al., 2006). The subtests from these assessments were utilized to evaluate cognitive functioning based on the Movement Disorder Society’s criteria for Level II cognitive assessment, encompassing five cognitive domains: attention and working memory, executive function, language, memory, and visuospatial abilities (see Table 1) (Litvan et al., 2012). Cognitive dysfunction was required to manifest as a consistent pattern in at least two subtests to be considered significant. We also used the Geriatric Depression Scale to assess the presence of depression (ranged 0–30) or depression symptoms severity (Montorio and Izal, 1996).

**Table 1:** Cognitive processes and theirs measures.

Cognitive process	Subtests from neuropsychological assessment
Attention and working memory	TMT (index B/A); Digit Span Forward from WAIS-R; Digit Span Backwards from WAIS-R; Serial Subtraction from MMSE;
Executive function	Phonological Verbal Fluency (response generation and ability to set maintenance of task rules); Ability to organize memorized information in CVLT; The index of recognized and spontaneously recalled words in CVLT;
Language	General communication skills (language expression) during interview; Language comprehension in different tasks; Category fluency task; Naming task in MMSE;
Memory	List of words in CVLT learning; List of words in CVLT delayed recall; Recognition of words in CVLT; ROCF delay recall;
Visuospatial function	ROCF copy; Drawing pentagon in MMSE; TMT A Visual Field Search Rate (independent from bradykinesia, assessed qualitatively);

MMSE, Mini-Mental State Examination; CVLT, Californian Verbal Learning Test; WAIS-R, Digit Span from Wechsler Intelligence Test; VFT, Verbal Fluency Test; TMT, Trail Making Test from Halstead-Reitan Battery; ROCF, Rey Osterrieth Complex Figure Test.

b2. Experiment ROBBIA (ROtman-Baycrest Battery for Investigation Attention)

The study used four tasks from the ROBBIA battery (Stuss et al., 2005; Stuss and Alexander, 2007). The aim was to measure reaction times (RT) in the three conditions: (1) simple reaction time (Simple RT), (2) choice reaction time (Choice RT), and (3) prepare reaction time consisting of two tasks (Prepare RT). Each participant was introduced to detailed instructions and performed a practice trial before starting the task. A 22-inch monitor and designed two-buttoned panel, created by the study’s author, was used for the reaction time measurement. The panel was designed as user-friendly even for participants with significant severity of motor disorders (the diameter of buttons was 2.5 cm). The button on the left was marked as button 1, and the button on the right was marked as button 2 (see Supplementary Figure 1). Pressing the button caused the stimulus to disappear and initiated a new time interval, randomly determined by the program. The tasks were programmed using the free software PsychoPy (Peirce et al., 2019). The stimuli presented during the trials were displayed at various time intervals: 3 s, 4 s, 5 s, 6 s, 7 s (interstimulus interval – ISI), with the frequency of intervals being equal in each trial.

Simple reaction time – the task required participants to respond to the appearance of the letter “A,” which was presented at varying time intervals in the center of the screen. Participants were instructed to respond as quickly as possible by pressing button 1 upon each occurrence of the target stimulus. A total of 50 stimuli were presented during a single session.

Choice reaction time – the task involved pressing button 1 when the letter B appeared on the screen and pressing button 2 when any other letter (A, C, or D) appeared. A total of 60 stimuli were presented in one session.

(3) and (4) Prepare reaction time – this task consisted of two trials. It differed from the choice reaction time task by an additional cue (star), which appeared at constant intervals before the actual stimulus. In the third trial, the star appeared 1 s before the stimulus, while in the fourth trial, it appeared 3 s before the letters. Each session in tasks 3 and 4 consisted of 60 stimuli. The participant’s task was to press button 1 for the letter D and button 2 for A, B, C.

Four tasks comprise indicators of three processes forming anterior attentional system processes (Stuss et al., 2005; Harciarek et al., 2016). The hypothetical processes and their measurement indicator are presented in the Supplementary Table 2 (Table 2).

**Table 2:** ROBBIA subtests and tested processes (Stuss et al., 2005; Harciarek et al., 2016).

ROBBIA’s subtest	Variables	Hypothesized component process
Simple Reaction Time	Reaction time	Energizing
	Change in reaction time in relation to interstimulus intervals (ISI)	Monitoring
Choice Reaction Time	Reaction time (in relation to the Prepare RT task)	Energizing
	Change in reaction time in relation to ISI	Monitoring
	Total number of mistake	Monitoring
	Relation of false positive to false negative mistake	Task setting
Prepare Reaction Time	Reaction time (especially in relation to the Choice RT task)	Energizing
	Total number of mistake	Monitoring
	Relation of false positive to false negative mistake	Task setting

Energization was calculated as the mean of all reaction times participant obtained in one of the trials. Secondly, the mean reaction times for all participants assigned to PD-NCC, PD-MCI, and PDD were calculated. Energization was measured in simple, choice, and prepare reaction time tasks (tasks 1, 2, 3, and 4). The monitoring consisted of two indicators: change in reaction time in relation to ISI and total number of errors. Changes in reaction time in relation to ISI were divided into two categories: short ISI (time intervals of 3 s and 4 s) and long ISI (time intervals of 6 s and 7 s). In the next step, the means of trials with short ISI and long ISI were calculated (first for each participant, then for the four groups). The total number of errors was defined as the number of mistakes made by pressing the wrong button (button 1 or button 2). Task setting was measured as the false positive/false negative error ratio. A false positive error occurred when reacting to the target as if it were a non-target. A false negative error was defined as responding to the non-target as if it were a target.

### 2.3 Statistical analysis

We tested all three hypotheses by comparing four independent groups (CG, PD-NCC, PD-MCI, PDD). As indicators of measured variables often were non-normally distributed (e.g., reaction times, mistakes), we utilized non-parametric statistical methods. Specifically, one-way comparisons were performed using the Kruskal-Walli’s test, while ordinal logistic regression, including interaction terms, was applied for two-way comparisons. All analyses were conducted using R software, with “ggstatsplot” package (Patil, 2021) for one-way comparisons and “ordinal” package for logistic regressions (Christensen, 2023). A significance threshold of *p* < 0.05 was used in all tests. Data are published online: https://osf.io/6thdx/?view_only=b286882698d741c3aa66561d3d022b2f. Given small sample sizes, with PDD group standing out (*n* = 12), we conducted a sensitivity power analysis to establish the smallest effect size that can be reliably (*α* = 0.05, power > 0.80) examined with such a sample (Lakens, 2022). We conducted a Monte Carlo simulation with 5,000 samples, which showed that for *n* = 12 pairwise differences larger than *d* = 1.2 are sufficiently powered.

## 3 Results

### 3.1 The characteristic of study groups

From 2020 to 2023, 96 participants with Parkinson’s disease (45 with PD-NCC, 39 with PD-MCI, and 12 with PDD) and 46 participants from the control group were recruited, and data were analyzed. The demographic and clinical characteristics of the participants from the clinical samples and the comparison group were well-balanced, as shown in Table 3. The results of the pilot study, which were published separately, were included in the main analyses (Golińska et al., 2021).

**Table 3:** Demographic and clinical characteristics of participants.

Variable	PD-NCC (*n* = 45)	PD-MCI (*n* = 39)	PDD (*n* = 12)	CG (*n* = 46)	Comparison test
		*M* (*SD*) [min-max]	*M* (*SD*) [min-max]	*M* (*SD*) [min-max]	
Age	64.2 (8.0)	64.8 (9.4)	67.9 (6.2)	62 (8.0)	*F*_(3.138)_ = 1.92;
	[45–75]	[39–82]	[57–77]	[40–75]	*p* = 0.129
*n* (%)					
Gender [men]	20 (44.4%)	20 (51.3%)	8 (66.7%)	16 (34.8%)	*χ*^2^_(3)_ = 4.84
					*p* = 0.184
Education level					
Primary	1 (2.2%)	2 (5.1%)	0 (0%)	0 (0%)	*H*_(3)_ = 22.18
Vocational	2 (4.4%)	6 (15.4%)	5(41.7%)	2 (4.4%)	*p* < 0.001
Secondary	22 (48.9%)	18 (46.2%)	6 (50%)	14 (30.4%)	
Bachelor or engineer	4 (8.9%)	5 (12.8%)	0 (0%)	4 (8.7%)	
Higher (master’s and above)	16 (35.6%)	8 (20.5%)	1 (8.33%)	26 (56.5%)	
M (SD)					
Depression (GDS)	11.2 (7.0)	8.8 (6.2)	9.7 (4.5)	6.0 (4.4)	*F*_(3.138)_ = 6.03
	[0–27]	[0–25]	[3–17]	[0–21]	*p* < 0.001
PD duration	122.5 (76.4)	91.0 (76.9)	141.0 (120.0)	*–*	*F*_(2.93)_ = 2.35
	[9–324]	[13–384]	[18–408]		*p* = 0.101
LEDD	1.066.31 (616.13)	847.03 (498.38)	1.247.08 (642.72)	*–*	*F*_(2.92)_ = 2.35
	[255–2.600]	[40–2.160]	[200–2.600]		*p* = 0.101
UPDRS (total score)	52.2 (*25.0*)	52.38 (*23.4*)	60.7 (*24.9*)		*F*(2.92) = 0.53
	[4–131]	[17–107]	[26–96]	*–*	*p* = 0.590
Part 1	11.4 (6.9)	10.0 (5.6)	12.6 (4.4)		*F*(2.92) = 1.03
	[0–38]	[0–23]	[6–21]	*–*	*p* = 0.362
Part 2	11.6 (8.1)	10.5 (6.9)	11.0 (6.1)		*F*(2.92) = 0.26
	[0–44]	[0–29]	[2–21]	*–*	*p* = 0.769
Part 3	25.0 (11.8)	29.3 (12.1)	33.8 (14.9)		*F*(2.92) = 2.92
	[4–53]	[5–57]	[9–58]	*–*	*p* = 0.059
Part 4	4.2 (4.3)	2.7 (4.0)	4.2 (3.1)		*F*(2.92) = 1.51
	[0–16]	[0–13]	[0–11]	*–*	*p* = 0.226
*n* (%)					

PD-NCC, participants with Parkinson’s disease with normal cognitive condition; PD-MCI, participants with Parkinson’s disease with mild cognitive impairment; PDD, participants with Parkinson’s disease with dementia; CG, control group; LEDD, Levodopa Equivalent Daily Dose; PD, Parkinson’s disease. n = sample size; M = mean; SD = standard deviation; min - minimum value; max - maximum value.

### 3.2 Differences in energization between PD-NCC, PD-MCI, PDD, and control group

**Figure 1:**
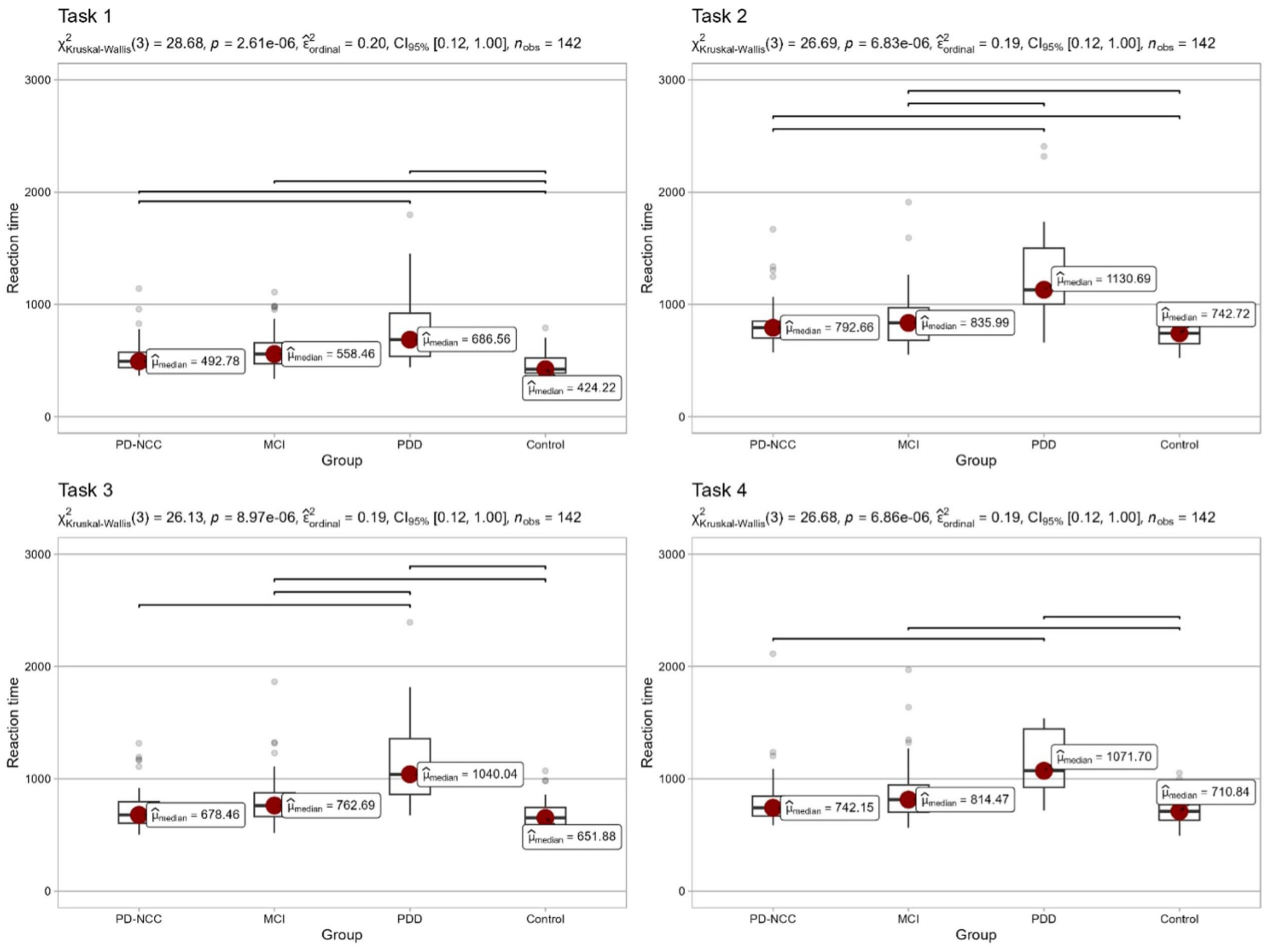
Energization comparisons across groups and tasks.

Both visual and formal comparisons of energization across groups are reported in Figure 1. Each of the four sections presents reaction times in one of the tasks that the participants took part in. For all pairwise comparisons we used Dunn’s nonparametric test, with Holm’s correction for multiple testing. Significant (*p* < 0.05) pairwise differences are marked in the figure. Detailed information concerning all pairwise tests is reported in the Supplementary Table 1.

Group comparison using the Kruskal-Wallis’s test revealed a statistically significant difference between CG and all clinical groups. Individuals from CG responded significantly faster in the simple reaction time (Task 1). Significant differences were observed between PD-NCC and PPD. However, no significant difference was found between PD-MCI and PD-NCC, as well as PD-MCI and PDD. In Choice Reaction Time (Task 2), reaction times increased in each group because of the decision-making process. Similarly to task 1, all clinical groups responded slower than CG. PD-NCC did not differ from PD-MCI. PDD performed significantly slower than other groups.

In Prepared Reaction Time with warning signal (Task 3) again, CG was the fastest reacting group. However, the profile of group differences was altered compared to previous findings. PD-NCC group did not respond significantly slower than CG. PD-MCI did not perform slower than PD-NCC but slower in comparison to CG. PDD was the slowest responding group.

In Prepared Reaction Time with warning signal (Task 4), PD-NCC did not differ from CG, but PD-MCI reacted slower than CG. PDD did not perform slower than PD-MCI, but the difference between medians can still be considered clinically valid.

Building on Stuss’s theoretical framework, which posits that the loss of the ability to sustain arousal for a duration of 3 s is essential for executing a rapid response to stimuli in task 4, and consequently facilitates faster reaction times in task 2, we conducted an exploratory analysis. This analysis involved the calculation of the reaction time difference (subtraction) between tasks 4 and 2, aiming to identify individuals exhibiting a specific pattern of energization deficits. The percentages of individuals with potential energization deficits are as follows: 29% in PD-NCC, 56% in PD-MCI, 41% in PDD but also 35% in control group.

### 3.3 Differences in monitoring between PD-NCC, PD-MCI, PDD, and control group

We found some differences between groups in terms of monitoring. Specifically, the number of mistakes was higher in PDD (Me = 3) and MCI groups (*Me* = 1) than PD-NCC (*Me* = 0) and Control group (*Me* = 0) in task 3, χ^2^_Kruskal-Wallis_(3) = 22.79; *p* < 0.01. In task 4, only PDD group (*Me* = 3) was different than PD-NCC or Control group (*Me* = 0 for both), χ^2^_Kruskal-Wallis_(3) = 13.31; *p* < 0.05. Moreover, we found no effects of ISI length on the reaction times in tasks 1 and 2, nor interactions of ISI length with a group (all *p*s > 0.66). We report average reaction times varied by ISI length in all groups in Table 4.

**Table 4:** Reaction times across different groups and varying ISI: tasks 1 and 2.

ISI	PD-NCC		PD-MCI		PDD		CG	
	*M*	*SD*	*M*	*SD*	*M*	*SD*	*M*	*SD*
Task 1								
Long	533.38	194.37	559.97	182.88	811.69	483.72	444.27	100.30
Short	561.20	140.86	610.04	205.91	791.85	303.00	473.97	106.22
Task 2								
Long	838.39	234.19	874.33	280.71	1243.15	449.93	733.11	114.16
Short	844.90	204.27	883.93	278.67	1380.86	676.71	736.24	113.53

Long ISI = 6 or 7 s; short ISI = 3 or 4 s; PD-NCC – participants with Parkinson’s disease with normal cognitive status, PD-MCI – participants with Parkinson’s disease with mild cognitive impairment; PDD – participants with Parkinson’s disease with dementia; CG – control group.

### 3.4 Differences in task-setting between PD-NCC, PD-MCI, PDD, and control groups

Finally, we found a single interaction effect concerning task-setting. Specifically, in the PDD group, the pattern of mistakes was different than in the other groups, as PDD participants made more positive than negative mistakes. We observed such results in all the tasks, although the interaction effect was significant only in task 4. In general, such a pattern indicates that PDD is prone to exhibit task-setting impairment (Table 5).

**Table 5:** Number of positive and negative mistakes across different groups.

Mistake type	PD-NCC		PD-MCI		PDD		Control	
	*M*	*SD*	*M*	*SD*	*M*	*SD*	*M*	*SD*
Task 2								
Negative	0.80	2.18	0.79	1.15	2.00	2.76	0.52	1.05
Positive	0.71	2.02	0.41	0.94	4.75	9.65	0.39	0.77
Task 3								
Negative	0.29	0.55	1.08	2.51	2.58	2.61	0.35	0.92
Positive	0.22	0.52	0.95	2.60	4.83	9.05	0.22	0.47
Task 4								
Negative	0.40	0.84	1.08	2.49	3.08	4.36	0.65	0.95
Positive	0.24	0.57	0.79	2.44	6.08	10.0	0.22	0.59

Positive mistake, reaction to non-target stimulus; Negative mistake, no reaction to target stimulus; PD-NCC, participants with Parkinson’s disease with normal cognitive status; PD-MCI, participants with Parkinson’s disease with mild cognitive impairment; PDD, participants with Parkinson’s disease with dementia; CG, control group.

## 4 Discussion

Study results suggest that the association between three cognitive statutes: PD-NCC, PD-MCI, PDD, and the Anterior Attentional System performance is not as gradual as we expected.

Concerning the first research question on differences in energization between PD-NCC, PD-MCI, PDD, and the control group, surprisingly, the most widespread frontal energization deficits were observed in PD-MCI and were not impaired in PD-NCC. It might be explained by the fact that levodopa treatment for Parkinson’s disease (PD) is effective in managing motor symptoms, while the disease may not yet be at an advanced stage sufficient to induce cognitive impairment. Therefore, no cognitive problems, even specific to frontal regions, have been noted. This is an interesting finding in the context of the ongoing debate regarding executive functioning appearing at the beginning of the disease, shortly after being diagnosed with Parkinson’s disease. Some studies indicated that executive problems may occur independently from mild cognitive impairment. Consequently, executive difficulties can be observed in the early stages of Parkinson’s disease and may even serve as a prodromal symptom of the condition (Muslimović et al., 2009; Dirnberger and Jahanshahi, 2013). We did not observe any statistical differences between each groups’ reaction times, which indicate no specific pattern in any of them. Thus, no energization deficits could be inferred. However, there were individuals with specific energization deficits across all the groups. It should be considered that the original research on energization deficits was conducted on individuals with selective frontal lobe damage (Stuss et al., 1995, 2005; Stuss and Alexander, 2007). Energization problems were also noted in patients with end-stage kidney disease (Harciarek et al., 2016), which suggests that energization deficits may be diagnosed selectively in somatic diseases. Assessing cognitive functions in individuals diagnosed with Parkinson’s disease and their comorbidities should be taken into account. It is hypothesized that the executive decline observed in individuals with normal cognitive status, as reported in previous studies, may be attributed to the fact that executive dysfunction is a common cognitive impairment across a range of medical conditions, including cardiovascular diseases (Rostamian et al., 2015; Jackson et al., 2021). Therefore, it might not be a direct effect of Parkinson’s disease itself, but rather the cumulative impact of Parkinson’s disease and associated comorbidities on overall health. Another explanation of executive problems depletion is that depression and anxiety may influence cognitive functioning (Alves et al., 2014). Thus, it is essential to control for this factor in studies examining similar phenomena. Additionally, the reaction time analysis across different tasks gives us a view into attentional and executive processes in individuals with PD. Already in the 80s, it was noticed that individuals with PD without cognitive impairment were able to utilize warning signals in reaction time tasks effectively (Bloxham et al., 1987). It also has been shown that the slower reaction time observed in individuals with PD mainly concerns simple reaction time. The reaction time in choice tasks without a warning signal remains a subject of debate. While some studies have reported slower reaction times in such tasks, the findings are not consistent across all research.

This study also aimed to assess differences in monitoring in PD-NCC, PD-MCI, PDD, and control groups. The study suggests that monitoring is preserved in PD-NCC and with PD-MCI but disrupted in PDD. A tendency to make more errors in the choice task was also observed among individuals with mild cognitive impairment. According to Stuss and Alexander (Stuss et al., 2005; Stuss and Alexander, 2007), individuals with monitoring decline make all kinds of errors, and participants with task-setting impairment present a tendency to make false-positive errors. The results of further analyses indicate that individuals with dementia tend to make false-positive errors. Thus, both indicators are not exclusive. The only group in which a characteristic pattern for monitoring impairment appears, characterized by an increase in reaction time with the lengthening of the interval between stimuli, is also the PDD group. However, this pattern was observed only in the simple task, which contradicts the assumption proposed by Stuss et al. (2005) and Stuss and Alexander (2007) that difficulties in task-setting are more prevalent when task difficulty increases. Possibly, the choice task involves different processes. Although the choice task is generally more challenging, the subprocesses involved in this task may be well preserved in dementia and differ from those involved in the simple reaction time. These hypotheses may include selective attention or response inhibition, which also might be involved in temporal monitoring. According to Stuss and Alexander (2007), a characteristic reaction time pattern in response to varied interstimulus intervals (ISI) appears in individuals with the lateral part of the right prefrontal cortex due to reduced time monitoring. Vallesi et al. (2007) obtained a similar reaction time pattern; however, with a shorter ISI, this suggests that the reaction pattern is more important than the length of ISI. Another study conducted among individuals with Parkinson’s disease implies that processing time intervals require efficient dopaminergic pathways for voluntary activities (Jurkowski et al., 2005). The right prefrontal cortex (monitoring neuroanatomical substrate) is rich in dopaminergic pathways. Thus, it has been assumed that part of the prefrontal cortex may play a crucial role in processing temporal information (Stuss and Alexander, 2007). However, another study investigating the neuroanatomical basis for estimating the duration of time intervals has revealed that even simple tasks involve coordinating many brain structures, such as the parietal areas and insula (Lewis and Miall, 2003). Therefore, we hypothesized that (1) monitoring impairment may be related to the selective time estimation problem and (2) two monitoring indexes, errors, and reaction times related to ISI might be independent processes.

The third research question was also not answered, which anticipated task-setting difficulties in PD-NCC, PD-MCI, PDD. The only group with task-setting deficits was PDD. Stuss and colleagues assumed task-setting is particularly important when starting activities that are not yet automated and, therefore, require conscious cognitive control (Stuss et al., 2005). According to Koerts et al. (2011), planning ability is well-preserved in individuals with Parkinson’s disease and allows them to compensate when performing tasks that require multitasking effectively. However, executive and attentional deficits are well-established in Parkinson’s disease, making the use of such “umbrella” terms for describing the cognitive functioning of individuals with PD, likely inappropriate (Kudlicka et al., 2011). It is more valuable to identify specific resources and skills that are deficient within each overarching category or “umbrella” term.

Summarizing, the Anterior Attentional System is surprisingly well-preserved in PD-NCC and PD-MCI; however, it is notably disturbed in PDD. A slight impairment in energization was observed across all cognitive statuses. Monitoring and task-setting processes were disrupted only in individuals with PDD, while these functions remained intact in those with PD-NCC and PD-MCI. Those results may suggest that the isolated and accurate measurement of attentional processes indicates that it is not as impaired as reported in other studies, which used non-experimental procedures, but rather paper-pencil neuropsychological tests.

### 4.1 Limitations

Parkinson’s disease is simultaneously an excellent but challenging starting point for studying mechanisms related to attention and executive processes due to the heterogeneity of the disease symptoms, which may influence the results and their interpretation. The following issues may somewhat limit these findings. First, we included a higher percentage of individuals with higher education in the comparison group compared to the participants with dementia. Additionally, lower education in the PDD group raises the question about the relationship between education level and risk of dementia development. So far, a systematic review and meta-analysis have shown a link between a lower education level and a higher likelihood of developing dementia (Caamaño-Isorna et al., 2006). Perhaps this explains difficulties in achieving similar education levels in all groups. Second, the methodology of anterior attentional processes examination needs to be more ecological. Although the use of ROBBIA may help with specific process distinction, it remains challenging to infer how the results from the experiment may be translated into the patient’s daily living functioning. To the best of the author’s knowledge, the ROBBIA has not been correlated with activities of daily living. Fifth, these findings may be somewhat limited by the sample size, which increases false negative error probabilities. Individuals with dementia often withdraw from undertaking intellectual challenges, which affects the recruitment efficiency in the current study.

### 4.2 Future directions

It is proposed that further exploration of the factors associated with energization deficits among individuals with Parkinson’s disease is proposed to identify the correlates of energization difficulties. Additionally, it would be beneficial to link the challenges noted in the experimental measurement with the problems experienced by patients in their everyday lives. It is worth continuing research on the ROBBIA methodology, as there are still interpretative ambiguities and measurement issues, such as linking attention to executive functions theory. Interestingly, Luria (1979) pointed out the possibility of paradoxical and pathological enhancement of the orienting reflex in individuals with frontal lobe damage. He did not assume “the ceiling effect” of the attention process but rather the optimum for each process and the risk of pathology resulting from compensatory processes. This perspective seems interesting in the context of anterior attentional system functioning. The following question might be asked: is it possible that any of the processes comprising AAS could take on a hyperactive, pathological form? For example, an overly active task-oriented process could lead to attention disorders (cognitive rigidity), generating a tendency to focus only on a selected type of stimulus. More research on this issue is required.

## Data Availability

The datasets presented in this study can be found in online repositories. The names of the repository/repositories and accession number(s) can be found in the article/Supplementary material.
