# Supplementary material for: Anterior attentional system efficacy in Parkinson’s disease: a cross-sectional study in Poland
Source: Front Hum Neurosci. 2025 Nov 13;19:1695299. doi: 10.3389/fnhum.2025.1695299 (PMC12657388; doi:10.3389/fnhum.2025.1695299)
Supplement: Supplementary file 1 [file Data_Sheet_1.docx]

Supplementary Material

# Supplementary Figure


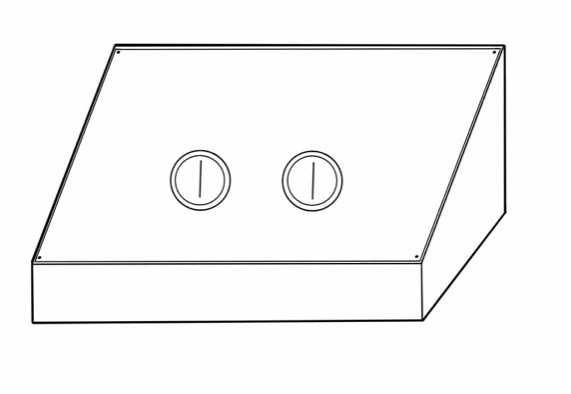


**Supplementary Figure 1.** Two-buttoned panel used in the study.

**2. Supplementary analyses**

2.1 Group comparisons with the smallest group (PDD, *n* = 12) omitted

2.1.1 Energization

As reported in Supplementary Figure 2, we found significant between-group differences in reaction times across tasks. In tasks 1 and 2 reaction times in control group were significantly lower than in PD-NCC and MCI groups but no significant differences emerged for the latter two groups. In tasks 3 and 4 control group reaction times were significantly lower only from MCI group, with PDD-NCC placed in the middle and thus not significantly different from any of the remaining two.


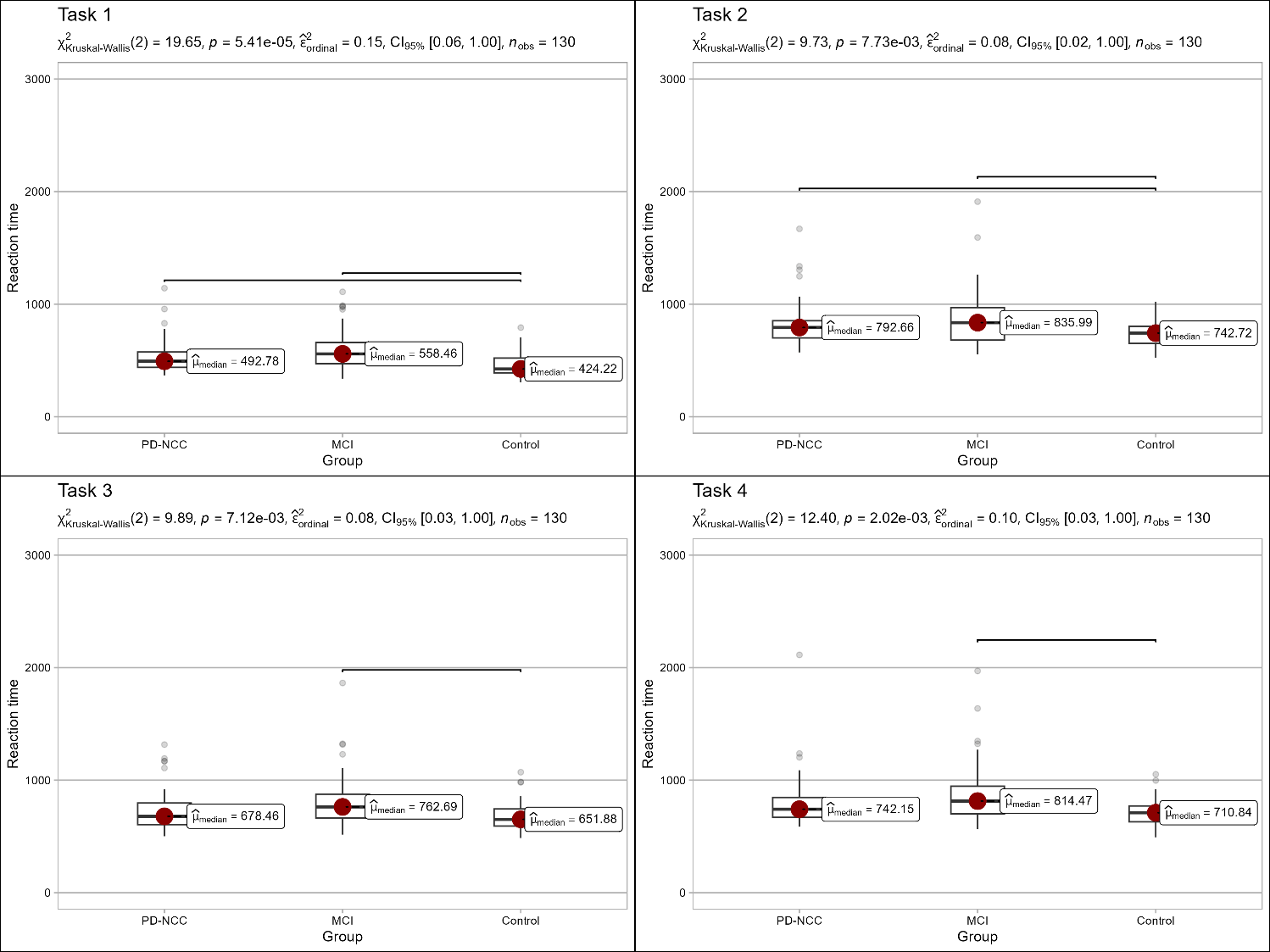


**Supplementary Figure 2.** Between-group comparisons of reaction times in different tasks. PDD group omitted.

2.1.2 Monitoring

The differences between groups in terms of mistakes that we reported in the paper were largely driven by PDD group. Analyses without that group are in line with those observations, as we did not find any differences in task 2, χ^2^_Kruskal-Wallis_(2) = 0.89; *p* < .640, significant difference between MCI (*Me* = 0) and two other groups (*Me* = 0 for both) in task 3, χ^2^_Kruskal-Wallis_(2) = 8.68; *p* = .01, and no differences for task 4, χ^2^_Kruskal-Wallis_(2) = 4.00; *p* = .14.

Ordinal regression analyses for ISI provided similar results with and without PDD group involved as a dummy. Specifically, we found no ISI length effect nor interaction effects between ISI length and group.

2.1.3 Task setting

Similar to the effects of number of mistakes, used to compare groups in terms of monitoring, the differences in pattern of mistakes, used to compare groups in terms of task setting, were driven by the PDD group. Consistent with the results reported in the main paper, ordinal regression analyses for the remaining three groups showed no differences between them, nor interactions with the type of mistake made (all *p* > .55).

2.2 Pairwise comparisons

All pairwise comparisons were made with Dunn’s test, adjusting the significance for multiple testing with Holm’s correction. Cohen’s *d* values were calculated as effect size measures to align with the Monte Carlo simulation power analyses.

**Supplementary Table 1**Pairwise comparisons for Kruskal-Wallis tests reported in the main paper

| Variable | group 1 | group 2 | *n*1 | *n*2 | *Z* | *p* | *p*-adj (Holm) | *d* |
| --- | --- | --- | --- | --- | --- | --- | --- | --- |
| ***Energization*** | | |  |  |  |  |  |  |
| Task 1 | NCC | MCI | 45 | 39 | 1.40 | 0.162 | 0.258 | -0.34 |
| Task 1 | NCC | PDD | 45 | 12 | 2.48 | 0.013 | 0.039 | -1.17 |
| Task 1 | NCC | Control | 45 | 46 | -2.95 | 0.003 | 0.013 | 0.64 |
| Task 1 | MCI | Control | 39 | 46 | -4.24 | 0.000 | 0.000 | 0.95 |
| Task 1 | PDD | MCI | 12 | 39 | -1.52 | 0.129 | 0.258 | 0.82 |
| Task 1 | PDD | Control | 12 | 46 | -4.39 | 0.000 | 0.000 | 1.73 |
|  |  |  |  |  |  |  |  |  |
| Task 2 | NCC | MCI | 45 | 39 | 0.26 | 0.796 | 0.796 | -0.16 |
| Task 2 | NCC | PDD | 45 | 12 | 3.36 | 0.001 | 0.004 | -1.56 |
| Task 2 | NCC | Control | 45 | 46 | -2.52 | 0.012 | 0.023 | 0.63 |
| Task 2 | MCI | Control | 39 | 46 | -2.69 | 0.007 | 0.022 | 0.71 |
| Task 2 | PDD | MCI | 12 | 39 | -3.13 | 0.002 | 0.007 | 1.26 |
| Task 2 | PDD | Control | 12 | 46 | -5.00 | 0.000 | 0.000 | 2.24 |
|  |  |  |  |  |  |  |  |  |
| Task 3 | NCC | MCI | 45 | 39 | 1.86 | 0.062 | 0.125 | -0.38 |
| Task 3 | NCC | PDD | 45 | 12 | 3.91 | 0.000 | 0.000 | -1.61 |
| Task 3 | NCC | Control | 45 | 46 | -1.21 | 0.226 | 0.226 | 0.37 |
| Task 3 | MCI | Control | 39 | 46 | -3.04 | 0.002 | 0.009 | 0.71 |
| Task 3 | PDD | MCI | 12 | 39 | -2.61 | 0.009 | 0.027 | 1.11 |
| Task 3 | PDD | Control | 12 | 46 | -4.70 | 0.000 | 0.000 | 2.04 |
| Task 3 | NCC | MCI | 45 | 39 | 1.61 | 0.108 | 0.121 | -0.30 |
|  |  |  |  |  |  |  |  |  |
| Task 4 | NCC | PDD | 45 | 12 | 3.48 | 0.001 | 0.003 | -1.28 |
| Task 4 | NCC | Control | 45 | 46 | -1.88 | 0.060 | 0.121 | 0.49 |
| Task 4 | MCI | Control | 39 | 46 | -3.42 | 0.001 | 0.003 | 0.83 |
| Task 4 | PDD | MCI | 12 | 39 | -2.36 | 0.018 | 0.055 | 0.87 |
| Task 4 | PDD | Control | 12 | 46 | -4.70 | 0.000 | 0.000 | 2.52 |
|  |  |  |  |  |  |  |  |  |
| ***Monitoring*** | | |  |  |  |  |  |  |
| Total errors in task 2 | NCC | MCI | 45 | 39 | 0.08 | 0.935 | 1.000 | 0.10 |
| Total errors in task 2 | NCC | PDD | 45 | 12 | 1.64 | 0.102 | 0.508 | -0.89 |
| Total errors in task 2 | NCC | Control | 45 | 46 | -0.76 | 0.450 | 1.000 | 0.19 |
| Total errors in task 2 | MCI | Control | 39 | 46 | -0.81 | 0.418 | 1.000 | 0.18 |
| Total errors in task 2 | PDD | MCI | 12 | 39 | -1.56 | 0.119 | 0.508 | 1.09 |
| Total errors in task 2 | PDD | Control | 12 | 46 | -2.13 | 0.033 | 0.199 | 1.22 |
|  |  |  |  |  |  |  |  |  |
| Total errors in task 3 | NCC | MCI | 45 | 39 | 2.55 | 0.011 | 0.043 | -0.45 |
| Total errors in task 3 | NCC | PDD | 45 | 12 | 4.14 | 0.000 | 0.000 | -1.36 |
| Total errors in task 3 | NCC | Control | 45 | 46 | 0.19 | 0.847 | 0.847 | -0.06 |
| Total errors in task 3 | MCI | Control | 39 | 46 | -2.38 | 0.017 | 0.052 | 0.43 |
| Total errors in task 3 | PDD | MCI | 12 | 39 | -2.38 | 0.017 | 0.052 | 0.79 |
| Total errors in task 3 | PDD | Control | 12 | 46 | -4.02 | 0.000 | 0.000 | 1.35 |
|  |  |  |  |  |  |  |  |  |
| Total errors in task 4 | NCC | MCI | 45 | 38 | 1.91 | 0.057 | 0.170 | -0.36 |
| Total errors in task 4 | NCC | PDD | 45 | 12 | 3.45 | 0.001 | 0.003 | -1.57 |
| Total errors in task 4 | NCC | Control | 45 | 46 | 0.78 | 0.435 | 0.484 | -0.20 |
| Total errors in task 4 | MCI | Control | 38 | 46 | -1.17 | 0.242 | 0.484 | 0.30 |
| Total errors in task 4 | PDD | MCI | 12 | 38 | -2.11 | 0.035 | 0.138 | 1.02 |
| Total errors in task 4 | PDD | Control | 12 | 46 | -2.95 | 0.003 | 0.016 | 1.54 |

2.3 Comparisons adjusted for education

As Kruskal Wallis test does not directly enable to add covariates and alternatives would change the interpretation of the dependent variable, we conducted a two-step procedure to adjust the results for between-group differences in education. First, we used a linear regression model to residualise dependent variables (reaction times, number of mistakes) from education. Then we used Kruskal-Wallis test similar to the one reported in the main paper but on residualised scores of the DV. We report the results of the omnibus tests in Supplementary Table 2 and pairwise comparisons (Dunn’s tests with Holm’s correction) in Supplementary Table 3.

**Supplementary Table 2**Group comparisons adjusted for education differences

| Variable | n | H | df | p |
| --- | --- | --- | --- | --- |
| Task 1 | 141 | 19.60812 | 3 | 0.000 |
| Task 2 | 141 | 15.92203 | 3 | 0.001 |
| Task 3 | 141 | 16.80729 | 3 | 0.001 |
| Task 4 | 141 | 17.30893 | 3 | 0.001 |
| Total errors in task 2 | 141 | 3.366349 | 3 | 0.339 |
| Total errors in task 3 | 141 | 10.04546 | 3 | 0.018 |
| Total errors in task 4 | 141 | 10.43248 | 3 | 0.015 |

**Supplementary Table 3**Pairwise comparisons for Kruskal-Wallis tests adjusted for education

| Variable | group 1 | group 2 | n1 | n2 | *Z* | *p* | *p*-adj (Holm) | *d* |
| --- | --- | --- | --- | --- | --- | --- | --- | --- |
|  |  |  |  |  |  |  |  |  |
| Task 1 | NCC | PDD | 45 | 12 | 2.27 | 0.023 | 0.088 | -1.09 |
| Task 1 | NCC | MCI | 45 | 38 | 1.17 | 0.242 | 0.293 | -0.30 |
| Task 1 | NCC | Control | 45 | 46 | -2.29 | 0.022 | 0.088 | 0.54 |
| Task 1 | PDD | MCI | 12 | 38 | -1.45 | 0.146 | 0.293 | 0.75 |
| Task 1 | PDD | Control | 12 | 46 | -3.76 | 0.000 | 0.001 | 1.58 |
| Task 1 | MCI | Control | 38 | 46 | -3.37 | 0.001 | 0.004 | 0.80 |
|  |  |  |  |  |  |  |  |  |
| Task 2 | NCC | PDD | 45 | 12 | 3.10 | 0.002 | 0.008 | -1.42 |
| Task 2 | NCC | MCI | 45 | 38 | -0.16 | 0.872 | 0.872 | -0.06 |
| Task 2 | NCC | Control | 45 | 46 | -1.36 | 0.174 | 0.522 | 0.44 |
| Task 2 | PDD | MCI | 12 | 38 | -3.15 | 0.002 | 0.008 | 1.18 |
| Task 2 | PDD | Control | 12 | 46 | -3.99 | 0.000 | 0.000 | 1.95 |
| Task 2 | MCI | Control | 38 | 46 | -1.14 | 0.255 | 0.522 | 0.43 |
|  |  |  |  |  |  |  |  |  |
| Task 3 | NCC | PDD | 45 | 12 | 3.45 | 0.001 | 0.003 | -1.49 |
| Task 3 | NCC | MCI | 45 | 38 | 1.28 | 0.202 | 0.404 | -0.29 |
| Task 3 | NCC | Control | 45 | 46 | -0.68 | 0.496 | 0.496 | 0.21 |
| Task 3 | PDD | MCI | 12 | 38 | -2.53 | 0.011 | 0.045 | 1.02 |
| Task 3 | PDD | Control | 12 | 46 | -3.90 | 0.000 | 0.001 | 1.78 |
| Task 3 | MCI | Control | 38 | 46 | -1.93 | 0.053 | 0.160 | 0.49 |
|  |  |  |  |  |  |  |  |  |
| Task 4 | NCC | PDD | 45 | 12 | 3.11 | 0.002 | 0.009 | -1.15 |
| Task 4 | NCC | MCI | 45 | 38 | 1.35 | 0.178 | 0.356 | -0.25 |
| Task 4 | NCC | Control | 45 | 46 | -1.17 | 0.242 | 0.356 | 0.38 |
| Task 4 | PDD | MCI | 12 | 38 | -2.15 | 0.031 | 0.094 | 0.77 |
| Task 4 | PDD | Control | 12 | 46 | -3.87 | 0.000 | 0.001 | 2.13 |
| Task 4 | MCI | Control | 38 | 46 | -2.47 | 0.013 | 0.054 | 0.64 |
|  |  |  |  |  |  |  |  |  |
| Total errors in task 2 | NCC | PDD | 45 | 12 | 0.58 | 0.562 | 1.000 | -0.81 |
| Total errors in task 2 | NCC | MCI | 45 | 38 | -0.91 | 0.361 | 1.000 | 0.16 |
| Total errors in task 2 | NCC | Control | 45 | 46 | 0.85 | 0.397 | 1.000 | 0.11 |
| Total errors in task 2 | PDD | MCI | 12 | 38 | -1.18 | 0.239 | 1.000 | 1.02 |
| Total errors in task 2 | PDD | Control | 12 | 46 | -0.03 | 0.974 | 1.000 | 1.07 |
| Total errors in task 2 | MCI | Control | 38 | 46 | 1.73 | 0.084 | 0.503 | -0.11 |
|  |  |  |  |  |  |  |  |  |
| Total errors in task 3 | NCC | PDD | 45 | 12 | 3.04 | 0.002 | 0.014 | -1.20 |
| Total errors in task 3 | NCC | MCI | 45 | 38 | 1.48 | 0.139 | 0.279 | -0.35 |
| Total errors in task 3 | NCC | Control | 45 | 46 | 1.82 | 0.069 | 0.245 | -0.35 |
| Total errors in task 3 | PDD | MCI | 12 | 38 | -2.00 | 0.046 | 0.228 | 0.73 |
| Total errors in task 3 | PDD | Control | 12 | 46 | -1.87 | 0.061 | 0.245 | 1.15 |
| Total errors in task 3 | MCI | Control | 38 | 46 | 0.25 | 0.802 | 0.802 | 0.23 |
| Total errors in task 3 | NCC | PDD | 45 | 12 | 2.24 | 0.025 | 0.132 | -1.39 |
|  |  |  |  |  |  |  |  |  |
| Total errors in task 4 | NCC | MCI | 45 | 38 | -0.05 | 0.959 | 0.959 | -0.21 |
| Total errors in task 4 | NCC | Control | 45 | 46 | 2.29 | 0.022 | 0.132 | -0.48 |
| Total errors in task 4 | PDD | MCI | 12 | 38 | -2.23 | 0.025 | 0.132 | 0.98 |
| Total errors in task 4 | PDD | Control | 12 | 46 | -0.77 | 0.444 | 0.887 | 1.28 |
| Total errors in task 4 | MCI | Control | 38 | 46 | 2.24 | 0.025 | 0.132 | -0.01 |
